# Local regression and control of T1‐2 nasopharyngeal carcinoma treated with intensity‐modulated radiotherapy

**DOI:** 10.1002/cam4.1866

**Published:** 2018-11-08

**Authors:** Fen Xue, Dan Ou, Chaosu Hu, Xiayun He

**Affiliations:** ^1^ Department of Radiation Oncology Fudan University Shanghai Cancer Center Shanghai China; ^2^ Department of Oncology Shanghai Medical College Shanghai China

**Keywords:** boost irradiation, chemotherapy, local control, T1‐2 nasopharyngeal carcinoma

## Abstract

**Objective:**

To observe the local regression and control in T1‐2 nasopharyngeal carcinoma (NPC) patients treated with intensity‐modulated radiotherapy (IMRT) and to analyze the related influencing factors.

**Methods:**

Between January 2006 and June 2014, 247 consecutive T1‐2 NPC patients treated with IMRT were retrospectively analyzed, with 126 (51.0%) N0‐1 disease and 121 (49.0%) N2‐3 disease. Among them, 72.9% received platinum‐based chemotherapy. The prescribed dose to gross tumor volume was 66 Gy/30 fractions.

**Results:**

By the end of IMRT, the chemoradiotherapy (CRT) group had higher local complete response (CR) rate compared with IMRT alone group (92.2% vs 74.6%, *P* < 0.001), but no significant difference was discovered in 5‐year local control (LC) rate (95.1% vs 94.9%, *P* = 0.968). Of the rest 31 patients with residual nasopharyngeal lesions after IMRT, those received boost irradiation (67.7%) also showed no improvement in 5‐year LC rate compared with the observational group (95.0% vs 100.0%, *P* = 0.307). With a median follow‐up of 63 months, the estimated 5‐year LC rate for the whole group was 95.1% (T1 vs T2: 95.9% vs 94.7%, *P* = 0.186). Prognostic factors for LC were found neither in univariate nor in multivariate analysis. Advanced N stage was found to be the only adverse prognostic factor for all the other survivals.

**Conclusions:**

Excellent LC could be achieved in T1‐2 NPC treated with IMRT. The addition of chemotherapy may offer short‐term response benefit, but no significant LC benefit, so did boost irradiation. Attention should be attached to advanced N stage, the exploration of the recurrence‐related factors, and the necessities of the additional treatment.

## 1 INTRODUCTION

Nasopharyngeal carcinoma (NPC) is a common endemic malignancy in southern China with relatively high radiochemosensitivity.^1^, ^2^ In the era of 2‐dimensional radiotherapy (2DRT), the addition of chemotherapy to radiotherapy showed improved LC and survival outcomes and was confirmed as standard treatment for locally advanced NPC.^3^ However, rapid technical development of radiotherapeutic equipment was witnessed in the past decades. Intensity‐modulated radiotherapy (IMRT) was proved to have advantages over 2DRT both in dose distribution and in normal tissue protection.^4^ Kam et al^5^ found that in the comparison of 2DRT, 3DRT, and IMRT plans, T1 NPC patients with prescribed dose of 66 Gy received improved dose to 95% gross tumor volume (62.5 Gy in 2D‐RT and 3DCRT, and 68.7 Gy in IMRT) and had better sparing of normal tissues (such as parotid glands and temporomandibular joints) with IMRT. Several studies also reported improved locoregional control and reduced adverse effects with IMRT, especially in early T‐stage disease.^6^, ^7^, ^8^ Though chemotherapy may lead to higher short‐term local response rates after radiotherapy, the recurrence hazard for T1‐2 disease was low.^9^, ^10^ Considering the theoretical advantages of IMRT, whether the addition of chemotherapy was beneficial to local control (LC) in NPC patients with T1‐2 stage is controversial.

Furthermore, it was reported by Xu et al^11^ that 27.9% NPC patients with T1‐2N1 NPC had residual tumors at the end of radiotherapy. Boost irradiation was delivered to most of the residual tumors and 95.3% got complete response 3 months after radiotherapy. But it is not clear whether the LC could be improved by the boost irradiation. Besides, the short‐term tumor response to treatment was found to be a prognostic factor for disease control. Peng et al^12^ reported higher 3‐year failure‐free survival rates and overall survival (OS) rates in the non‐residual group compared with residual group, which may help to develop individualized treatment strategies. In this retrospective study, we aimed to observe the local regression and control in T1‐2 NPC patients treated with IMRT and to analyze the related influencing factors.

## 2 METHODS

### 2.1 Patient selection and treatment delivery

Between January 2006 and June 2014, consecutive patients with biopsy‐proven non‐metastatic T1‐2 NPC that was treated with IMRT at the Fudan University Shanghai Cancer Center were retrospectively enrolled. Written consent was obtained from all the patients, and the study was approved by the ethical review board of our institution. All patients completed a pretreatment evaluation, which included physical examination, biochemical and hematological blood tests, nasopharyngoscopy, pathology of the nasopharynx, nasopharyngeal and neck magnetic resonance imaging (MRI), chest X‐ray or computed tomography (CT), abdominal sonography or CT, and bone scintigram (for N2‐3 disease). Additional tests (such as Positron Emission Tomography‐Computed Tomography, puncture or biopsy for suspicious lesions in other organs) were performed in patients with suspicious findings. Patients were restaged according to the 7th edition of the American Joint Committee on Cancer (AJCC) staging system.

All patients were treated with IMRT. The gross tumor volume (GTV) included primary nasopharyngeal tumor and involved lymph nodes found in clinical and imaging examinations. The clinical target volume (CTV) included the nasopharynx, parapharyngeal space, retropharyngeal lymph node, posterior one‐third of the nasal cavity and maxillary sinus, anterior one‐third of the clivus, pterygoid plates, inferior sphenoid sinus, and drainage of the neck (levels II, III, and Va in patients with N0 stage and levels II‐Vb in patients with N1‐3 stage). The prescribed dose was 66.0 Gy to the planning target volume of the GTV in 30 fractions. The PTV60 (high‐risk CTV + 5.0 mm) was prescribed 60 Gy in 30 fractions. The PTV54 (low‐risk CTV + 5.0 mm) was prescribed 54 Gy in 30 fractions. All patients were treated 30 fractions following a routine schedule (one fraction daily for 5 days per week). Boost irradiation was offered for patients with residual disease at the attending physician's discretion. This additional radiation was delivered by intracavitary brachytherapy or external beam radiation.

All the patients with N0 stage were not administered chemotherapy. Most of the patients with N1 stage (those with the diameter of lymph node ≥3 cm)and all the patients with N2‐3 stage were administered platinum‐based chemotherapy except those with abnormal indexes who cannot tolerate chemotherapy. A total of 180 (72.9%) patients received chemotherapy, including induction chemotherapy ± concurrent chemotherapy or adjuvant chemotherapy. Concurrent chemotherapy consisted of cisplatin 30 mg/m^2^ weekly during IMRT. Regimens for induction chemotherapy and adjuvant chemotherapy included TPF (docetaxel 60 mg/m^2^/d, day 1, cisplatin 25 mg/m^2^/d, days 1‐3, and 5‐fluorouracil 0.5 g/m^2^/d with a 120‐h infusion), PF (cisplatin 25 mg/m^2^/d, days 1‐3, and 5‐fluorouracil 0.5 g/m^2^/d with a 120‐h infusion), and GP (gemcitabine 1 g/m^2^/d, day 1, day 8, and cisplatin 25 mg/m^2^/d, days 1‐3). Usually, IMRT was implemented 3 weeks after induction and adjuvant chemotherapy was administered 4 weeks after the completion of radiotherapy.

Radiation‐related acute toxicities were documented according to the Radiation Morbidity Scoring Criteria of the Radiation Therapy Oncology Group (RTOG). Patients were evaluated weekly during radiotherapy. Evaluation of short‐term tumor response was based on MRI according to the Response Evaluation Criteria for Solid Tumors version 1.1. After treatment completion, we did follow‐up every 3 months in the first 2 years, every 6 months in the following 3 years, and annually thereafter. Routine follow‐up included nasopharyngoscopy and physical examination. Nasopharyngeal and neck MRI were performed every 6‐12 months during follow‐up. Chest radiography and abdominal sonography were conducted annually. Further investigations were recommended when clinically indicated.

### 2.2 Statistical analysis

Local control was calculated from the date of initiation of treatment to the date of local failure or last follow‐up. OS was calculated from the date of initiation of treatment to the date of death or last follow‐up. Distance metastasis‐free survival (DMFS) was calculated from the date of initiation of treatment to the date of metastasis or last follow‐up. Progression‐free survival (PFS) was calculated from the date of initiation of treatment to the date of locoregionally failure, metastasis, death, or last follow‐up. The Kaplan‐Meier method was used to estimate the rates of LC, OS, DMFS, and PFS. The distributions of the survivals were compared using the log‐rank test. Univariate analysis was performed with the Cox proportional hazard model. Variables with a *P* value <0.2 by univariate analysis entered for the multivariate analysis. Clinically highly relevant variables (such as T and N stage) were included in multivariate analysis despite their *P* value >0.2 on univariate analysis. All statistical tests were two‐sided, and *P* < 0.05 was considered statistically significant. Statistical analyses were performed with SPSS 22.0 software (SPSS Inc, Chicago, IL, USA). Survival curves were generated using GraphPad Prism, version 7.0 (GraphPad Software, La Jolla, CA, USA).

## 3 RESULTS

### 3.1 Patient characteristics and survival

Between January 2006 and June 2014, 247 consecutive patients with T1‐2 NPC were treated at our center. The median age at diagnosis was 50 years (range, 16‐78 years). Of the 247 patients, 180 (72.9%) were treated with CRT and 67 (27.1%) were treated with IMRT alone. Demographic and clinical characteristics of the whole group of patients were summarized in Table 1.

**Table 1:** Demographic and clinical characteristics (n = 247)

Characteristic	No. of patients	Percent (%)
Age (y)		
≤50	130	52.6
>50	117	47.4
Gender		
Male	180	72.9
Female	67	27.1
Histology		
Non‐keratinizing	234	94.7
Others	13	5.3
Karnofsky performance status score		
90‐100	132	53.4
70‐80	115	46.6
T stage		
T1	84	34.0
T2	163	66.0
N stage		
N0‐1	126	51.0
N2‐3	121	49.0
PET/CT		
Yes	51	79.4
No	196	20.6
Treatment		
CRT	180	72.9
IMRT alone	67	27.1
Waiting time for radiotherapy (d)		
≤46	132	53.4
>46	115	46.6
Duration of radiotherapy (d)		
≤43	135	54.7
>43	112	45.3

CRT, chemoradiotherapy; IMRT, intensity‐modulated radiotherapy.

**Figure 1:**
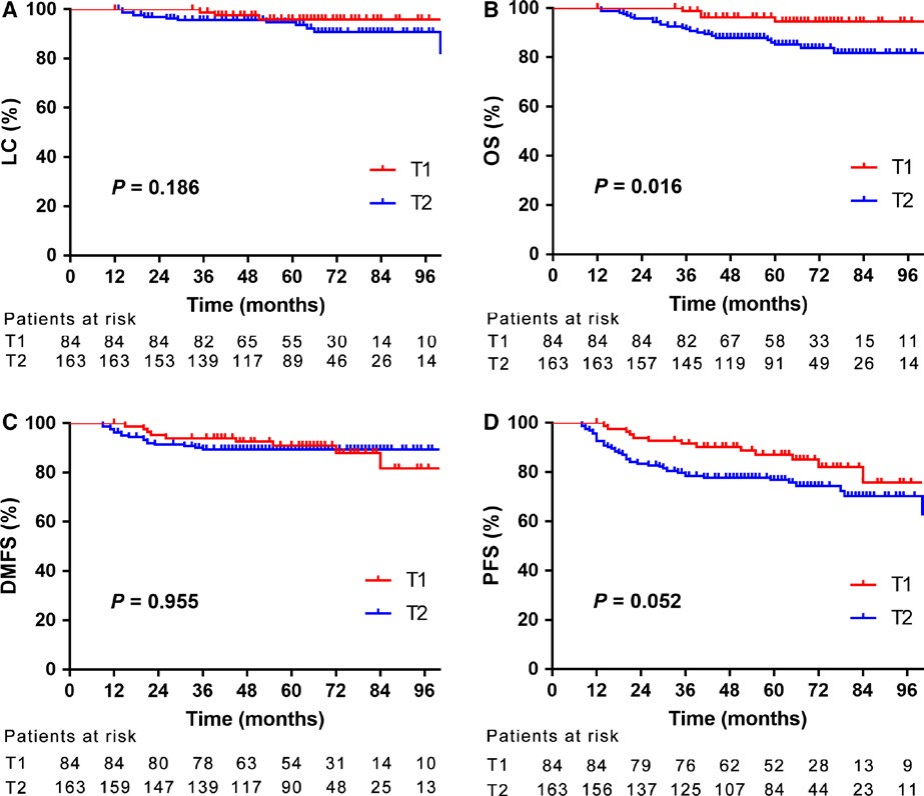
The estimated 5‐year local control (A), overall survival (B), distance metastasis‐free survival (C), and progression‐free survival (D) rates between patients with T1 and T2 stage, respectively

The median follow‐up period was 63 months (range 12‐145 months) for the whole group. A total of 51 patients experienced failures at different sites: local only 3.2%; regional only 4.9%; distant only 8.9%; local and regional only 2.4%; distant ± local ± regional 1.6%; and the distant site composed the most common failure. By the last follow‐up, the median time to local relapse was 35.0 months (range, 14.0‐101.0 months; Table 2). The estimated 5‐year LC rate for the whole group was 95.1%, which was 95.9% and 94.7% for patients with stage T1 and T2, respectively (*P* = 0.186, Figure 1A). The estimated 5‐year OS, DMFS and PFS were 88.4%, 89.9%, and 80.4%, which were 94.6% vs 85.2%, 90.9% vs 89.4% and 87.1% vs 76.9% for patients with stage T1 and T2, respectively (*P* = 0.016, *P* = 0.955 and *P* = 0.052, Figure 1B‐D).

**Table 2:** Details of patients developing local recurrence after treatment (n = 15)

No.	Stage	Recurrent time (mo)	Status
1	T1N0	51	Alive
2	T2N2	66	Alive
3	T2N1	101	Alive
4	T2N2	61	Dead
5	T2N2	64	Alive
6	T2N0	17	Alive
7	T1N3	39	Alive
8	T2N3	53	Dead
9	T1N1	35	Alive
10	T2N3	20	Dead
11	T2N1	17	Alive
12	T2N3	14	Alive
13	T2N3	14	Dead
14	T2N1	26	Alive
15	T2N3	29	Alive

### 3.2 Local regression and treatment outcomes

Of 180 patients in the CRT group, 177 patients received at least two cycles of IC, three patients discontinued IC after one cycle due to bone marrow suppression (grade 4) (2 patients) and liver function damage (grade 2) (1 patient). The response to treatments of the nasopharyngeal lesions was evaluated by MRI scans and nasopharyngoscopy. After IC, 14 (7.8%) patients developed local complete response (LCR), 136 (75.5%) patients developed local partial response (LPR) and 27 (15.0%) patients remained local stable disease (LSD). The rest 3 (1.7%) patients were not evaluated for their discontinuation of IC after one cycle. All patients completed the planned course of IMRT. In the CRT group, response rates were as follows after IMRT: LCR in 166 patients (92.2%) and LPR in 14 patients (7.8%). Ten of the LPR patients received boost irradiation at the primary site after the planned course of IMRT, with three treated with a brachytherapy boost (8 Gy) and seven treated with external beam irradiation (4.4 Gy/2 Fraction). In the IMRT alone group, response rates were as follows after IMRT: LCR in 50 patients (74.6%) and LPR in 17 patients (25.4%). Eleven of the LPR patients received boost irradiation at the primary site after the planned course of IMRT, with one treated with a brachytherapy boost (7 Gy) and 10 treated with external beam irradiation (4.4 Gy/2 Fraction). All the LPR patients developed LCR within 12 months after IMRT. Among them, 85.7% developed LCR within 6 months in the boost irradiation group and 80.0% in the observational group (*P* = 0.686).

Compared with the IMRT alone group, the CRT group had higher LCR rate after IMRT (92.2% vs 74.6%, *P* < 0.001, *χ* = 13.773), but no significant difference was discovered between the two groups in estimated 5‐year LC rate (95.1% vs 94.9%, *P* = 0.968). Of the patients with residual nasopharyngeal lesions after the planned course of IMRT, those received boost irradiation showed no improvement in estimated 5‐year LC rate compared with the observational group (95.0% vs 100.0%, *P* = 0.307).

### 3.3 Survival rates based on different N stage

**Figure 2:**
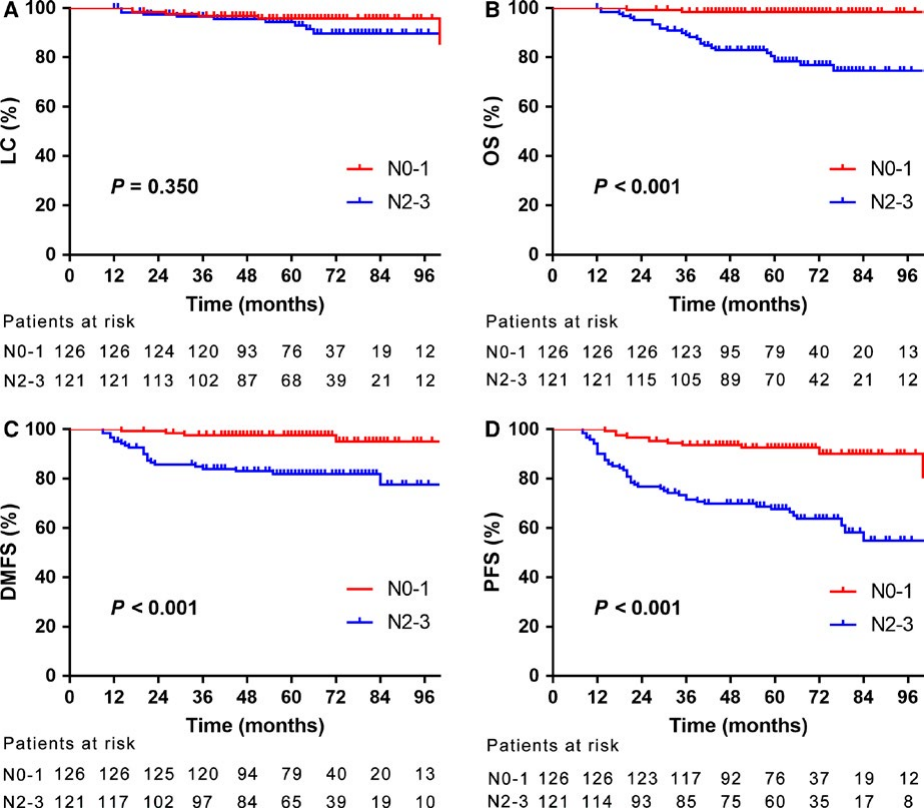
The estimated 5‐year local control (A), overall survival (B), distance metastasis‐free survival (C), and progression‐free survival (D) rates between patients with N0‐1 and N2‐3 stage, respectively

For the whole group, no significant difference was found in 5‐year LC rate (95.7% vs 94.4%, *P* = 0.350, Figure 2A) between N0‐1 and N2‐3 stage. While patients with N0‐1 stage showed improved 5‐year OS, DMFS and PFS compared with the rest patients (98.4% vs 78.3%, *P < *0.001; 97.6% vs 81.9%, *P < *0.001; 92.5% vs 67.7%, *P < *0.001; Figure 2B‐D). In patients with N1 stage, 77.5% received CRT and 22.5% received IMRT alone, but no significant differences for 5‐year LC, OS, DMFS or PFS rates were found between the two groups (96.7% vs 94.4%, *P = *0.149; 100.0% vs 100.0%, *P* > 0.050; 98.4% vs 100.0%, *P = *0.428; 95.1% vs 94.4%, *P = *0.497).

### 3.4 Acute toxicities

During radiotherapy, almost all hematologic adverse events were ≤grade 2. The most common non‐hematologic adverse events during radiotherapy were mucositis and weight loss. The rates of grade 1‐2, 3 and 4 mucositis were 142 (57.5%), 101 (40.9%), and 4 (1.6%), respectively. To relieve mucosal reaction, intravenous anti‐inflammation fluid infusion was administrated to 55.5% patients with median duration of 6 days (range, 2‐18 days). The median weight loss was 8.3% in all patients, and 156 (63.2%), 85 (34.4%), and 6 (2.4%) patients had grade 0‐1, 2, and 3 weight loss, respectively. The estimated 5‐year LC rate showed no difference among the patients with different levels of weight loss (94.4% vs 96.2% vs 100.0%, *P* = 0.469). No grade 5 acute toxicity occurred.

### 3.5 Prognostic factors

Table 3 summaries the prognostic factors for different survival rates in univariate and multivariate analysis. In this study, the absence of chemotherapy, boost irradiation, and T stage as well as the rest factors were not found to be related with the prognosis of LC either in univariate or in multivariate analysis. However, advanced age, poor performance status score, advanced N stage, and the absence of chemotherapy were found to be adverse prognostic factors for OS in multivariate analysis. Advanced N stage was also found to be adverse prognostic factors for DMFS and PFS.

**Table 3:** Univariate and multivariable Cox proportional hazard regression models for LC, OS, DMFS, and PFS

Variables	LC						OS					
	Univariate analysis			Multivariable analysis			Univariate analysis			Multivariable analysis		
	HR	95% CI	*P*	HR	95% CI	*P*	HR	95% CI	*P*	HR	95% CI	*P*
Age (y)			0.637						0.024			0.026
≤50	1						1			1		
>50	0.780	0.277‐2.193					2.490	1.126‐5.508		2.469	1.112‐5.479	
Gender			0.220						0.258			
Male	1						1					
Female	0.394	0.089‐1.745					0.572	0.217‐1.504				
Histology			0.133			0.111			0.802			
Non‐keratinizing	1			1			1					
Others	3.132	0.706‐13.900		3.393	0.755‐15.250		0.775	0.105‐5.708				
Karnofsky performance status score			0.750						0.007			0.019
90‐100	1						1			1		
70‐80	0.845	0.300‐2.382					3.123	1.374‐7.100		2.726	1.176‐6.319	
T stage			0.199			0.190			0.024			0.133
T1	1			1			1			1		
T2	2.293	0.646‐8.132		2.377	0.652‐8.663		3.389	1.176‐9.773		2.289	0.778‐6.734	
N stage			0.355			0.466			<0.001			<0.001
N0‐1	1			1			1			1		
N2‐3	1.629	0.579‐4.578		1.483	0.515‐4.271		14.422	3.423‐60.770		37.425	6.566‐213.333	
PET/CT			0.195			0.165			0.861			
Yes	1			1			1					
No	0.491	0.168‐1.440		0.461	0.155‐1.374		0.917	0.348‐2.414				
Treatment			0.968						0.129			0.004
CRT	1						1			1		
IMRT alone	1.024	0.326‐3.216					2.272	0.788‐6.551		8.748	1.971‐38.829	
Waiting time for radiotherapy (d)			0.544						0.021			0.266
≤46	1						1			1		
>46	1.369	0.496‐3.779					2.545	1.151‐5.628		1.745	0.654‐4.652	
Duration of radiotherapy (d)			0.607						0.950			
≤43	1						1					
>43	0.763	0.271‐2.145					1.024	0.487‐2.153				

Variables	DMFS						PFS					
	Univariate analysis		*P*	Multivariable analysis		*P*	Univariate analysis		*P*	Multivariable analysis		*P*
	HR	95% CI		HR	95% CI		HR	95% CI		HR	95% CI	
Age (y)			0.965						0.454			
≤50	1						1					
>50	0.983	0.454‐2.127					1.224	0.721‐2.078				
Gender			0.177			0.177			0.189			0.183
Male	1			1			1			1		
Female	0.480	0.165‐1.392		0.479	0.165‐1.393		0.642	0.332‐1.244		0.638	0.329‐1.237	
Histology			0.441						0.682			
Non‐keratinizing	1						1					
Others	0.046	0.000‐116.921					0.744	0.181‐3.055				
Karnofsky performance status score			0.096			0.111			0.088			0.105
90‐100	1			1			1			1		
70‐80	1.962	0.888‐4.335		1.922	0.860‐4.296		1.592	0.932‐2.719		1.568	0.910‐2.703	
T stage			0.956			0.527			0.056			0.220
T1	1			1			1			1		
T2	1.023	0.456‐2.296		0.767	0.338‐1.743		1.835	0.985‐3.420		1.488	0.789‐2.804	
N stage			0.001			0.010			<0.001			<0.001
N0‐1	1			1			1			1		
N2‐3	6.220	2.143‐18.053		5.396	1.498‐19.434		4.860	2.509‐9.415		6.477	2.722‐15.411	
PET/CT			0.991						0.561			
Yes	1						1					
No	1.006	0.379‐2.671					0.827	0.435‐1.570				
Treatment			0.039			0.665			0.058			0.036
CRT	1			1			1			1		
IMRT alone	0.219	0.052‐0.928		1.527	0.225‐10.356		0.501	0.245‐1.023		3.058	1.078‐8.671	
Waiting time for radiotherapy (d)			0.003			0.100			0.006			0.152
≤46	1			1			1			1		
>46	4.026	1.617‐10.028		2.455	0.842‐7.156		2.167	1.250‐3.754		1.632	0.834‐3.193	
Duration of radiotherapy (d)			0.946						0.920			
≤43	1						1					
>43	1.027	0.475‐2.220					0.973	0.572‐1.655				

LC, local control; OS, overall survival; DMFS, distant metastasis‐free survival; PFS, progression‐free survival.

## 4 DISCUSSION

In the current study, we retrospectively analyzed local regression and control of NPC patients with T1‐2 stage. They all received IMRT with or without platinum‐based chemotherapy. The 5‐year LC was 95.1% and no significant difference was found between T1 and T2 disease (95.9% vs 94.7%, *P* = 0.186). Consistent with the literatures, excellent LC could be achieved for T1‐2 NPC using IMRT.^8^, ^13^ Therefore, the necessities and efficacy of the additional treatment were controversial.

Various randomized studies showed that the application of platinum‐based chemotherapy was beneficial to the tumor response and locoregional control of the locally advanced NPC.^14^, ^15^ Also, the total doses of cisplatin delivered during the CRT have been proved to be related with locoregional control.^16^, ^17^ However, most of the studies above were based on the 2DRT. With the advantages of conformal dose distribution and normal tissue protection, IMRT has replaced conventional radiotherapy as the standard radiotherapy treatment for NPC in the centers where the equipment was available.^5^, ^18^ Randomized controlled trials showed improved LC and reduced radiation‐related toxicities with IMRT compared with 2DRT, especially in patients with early disease.^1^, ^19^ Sun et al^13^ reported long‐term outcomes of IMRT for NPC. They found that when IMRT was used for NPC patients, the application of concurrent chemotherapy failed to improve the LC or prognosis but increased the severity of acute toxicities. So, they believed that IMRT may “counteract” the effect of concurrent chemotherapy on LC improvement. It was also reported that cisplatin may increase the radiosensitivity of parotid glands to cause a higher probability of parotid gland tissue damage.^20^ Considering the excellent LC achieved in the T1‐2 NPC (5‐year LC rate >90%),^8^, ^13^ the additional chemotherapy seemed to be unnecessary. However, Luo et al^20^ demonstrated that the addition of concurrent chemotherapy to IMRT resulted in 18.2% increases in 3‐year local recurrence‐free survival (*P* < 0.05) for T1‐2 NPC patients and was established as a favorable prognostic factor for local recurrence‐free survival in the multivariate analysis. For T1‐2 NPC patients in this study, the CRT group had higher LCR rate after IMRT (92.2% vs 74.6%, *P* < 0.001, *χ* = 13.773), but no significant difference was discovered between the two groups in estimated 5‐year LC rate (95.1% vs 94.9%, *P* = 0.968). The higher local response rate has not brought LC benefit. It seemed the demand for the addition of chemotherapy depended more on the status of positive lymph nodes than on the primary site in T1‐2 disease.

By the time of the end of IMRT, the necessity of the additional boost irradiation for T1‐2 disease with residual tumors is controversial, especially when the prescribed dose is considered sufficient. In the era of 2DRT, several retrospective studies found that additional boost irradiation was an excellent method of enhancing LC for patients with NPC with early T1‐2 disease.^21^, ^22^ However, high incidence of late toxicities was also reported by Schinagl et al^22^ with additional boost irradiation, which suggested an overtreatment. Meanwhile, other studies showed no improvement of LC and survival outcomes with boost irradiation for patients with persistent primary tumor at the end of radiotherapy.^23^, ^24^ IMRT was known to harbor different dosimetric characteristics from 2DRT, but the controversy still existed. Chao et al^25^ demonstrated improved LC with boost irradiation for T1 disease treated with IMRT while Ou et al^26^ believed no survival benefits for persistent disease can be reached by boost irradiation when using IMRT. They suggested that IMRT may “counteract” the effect of boost irradiation on LC improvement and the residual tumor after intensive chemoradiation may suggest a more treatment‐reluctant phenotype. In this study, of the patients with residual primary tumor after the planned course of IMRT, those received boost irradiation showed no improvement in estimated 5‐year LC rate compared with the observational group (95.0% vs 100.0%, *P* = 0.307). Besides, spontaneous regression was found in most of the residual tumor, as reported by Xu et al.^11^ We found 80.0% of the residual tumor disappeared spontaneously within 6 months after IMRT in the group without boost irradiation, similar to the 85.7% in the boost irradiation group (*P* = 0.686). All the residual lesions disappeared within 12 months after IMRT. Therefore, the benefit of boost irradiation in IMRT needs to be reevaluated.

Retrospective study found that the majority of local recurrence occurred within 5 years.^9^ Similar to the literature, our data showed that 14 patients (93.3%) developed recurrence within 66 months, only 1 patient (6.7%) occurred after 8 years. Long‐term follow‐up is still essential after 5 years. For T1‐2 NPC, the influencing factors for local recurrence were still unknown. In our multivariate analysis, none of the factors was found to be related with the prognosis of LC. But advanced N stage was found to be the only adverse prognostic factor for both OS and DMFS, as well as PFS. Therefore, the local recurrence‐related factors need to be further explored, but more attention needs to be payed for patients with advanced N stage.

Most of the toxicities in this study were mild. Mucositis and weight loss accounted for the most common acute toxicities during radiotherapy. Among them, 42.5% and 2.4% patients developed grade 3 more mucositis and weight loss, respectively. Previous study showed that weight loss was associated with an unfavorable prognosis.^27^ However, the estimated 5‐year LC rate in this study showed no difference among the patients with different levels of weight loss (grade 0‐1:94.4% vs grade 2:96.2% vs grade 3:100.0%, *P* = 0.469). We suggested that most T1‐2 disease were limited to the nasopharynx or the nearby structures, so the structural changes induced by weight loss may have little effect on LC in T1‐2 NPC. There are several limitations for this study. First, selection bias was unavoidable owing to its retrospective nature. Second, the emphasis of this study was on the analysis of local regression and control for T1‐2 NPC, so the N stage‐related data were not in further discussion. Third, the small sample size of patients treated with IMRT alone meant we could not fully assess the predictive effect of additional chemotherapy.

In conclusion, IMRT provided excellent LC for T1‐2 NPC, but small part of patients still suffered from recurrence. Our study showed that the addition of chemotherapy conferred improved short‐term local response. However, the short‐term benefits had not been transferred into long‐term LC benefits. Also, additional boost irradiation showed no LC benefits. Clinicians should be cautious about the addition of chemotherapy and boost irradiation, considering the potential increased adverse effects. Besides, advanced N stage was found to be the only adverse prognostic factor for both OS and DMFS, as well as PFS. We should attach more attention to patients with advanced N stage in the future study. Further researches are needed to explore the recurrence‐related factors and the necessities of the additional treatment.

## CONFLICT OF INTEREST

None declared.
